# Proceedings of Veterinary Medical Societies

**Published:** 1887-10

**Authors:** 


### PROCEEDINGS VETERINARY MEDICAL SOCIETIES.

I. NEW JERSEY STATE VETERINARY SOCIETY.—Twenty-four *graduates*, fifteen of whom had been members of the old association, have assisted in organizing a NEW JERSEY STATE VETERINARY SOCIETY to which *only graduates, in good standing, from some Veterinary College or University having power by law to grant diplomas, are eligible to membership.* Every graduate known in the State was duly notified of the action about to be taken and a meeting was held August 4th, 1887, at the office of Dr. William Herbert Lowe in Patterson, New Jersey, where officers were elected and the organization completed. The society proceeded immediately to comply with the term and provisions of the act of the Legislature for the promotion of veterinary science and art. The officers and members present signed and sealed the certificate of incorporation which had been previously drawn up by Senator Griggs, after which the document was forwarded to the Secretary of State at Trenton, as required by law. Six of the incorporators are graduates of the *American Veterinary College*, three are graduates of the *Columbia Veterinary College*, two are graduates of the *Ontario Veterinary College*, two are graduates of the *Chicago Veterinary College* and one is a graduate of the *Royal Saxon College.*

The following are the officers elected at the meeting held in Patterson, August 4th :

*President*, J. C. Corlies, D. V. S , of Newark, N. J.

*First Vice-President*, Edwin R. Voorhees, D.V.S., of Somerville, N. J.

*Second Vice-President.* Eldon L. Loblein, D. V. S., of New Brunswick, N. J.

*Secretary.* William Herbert Lowe, D. V. S., of Paterson, N. J.

*Treasurer.* Ludwig R. Sattler, D. V. S., of Newark, N. J.

*Censors.* Andrew Shirk, V. S., of Newark ; Joseph Naylor, D. V. S., of Jersey City ; Elmore R. Mercer, D. V. S., of Montclair ; Matthew A. Pierce, D. V. S., of Paterson ; and Robert W. Carter, V. S., of Jobstown.

All except two of the newly elected officers had been members of the old association. They had disconnected themselves with it simply because they did not consider that it would be advantageous to them or to the veterinary profession of New Jersey to be associated with the non-graduates who were members. It appears that recently the non-graduates have taken a more active part in the affairs of the association than the graduates, for at a recent meeting it is alleged that four of the officers elected were non-graduates.

The following distinguished gentlemen were proposed by the secretary, for honorary membership, in the NEW JERSEY STATE VETERINARY SOCIETY.

Professor Liautard, Dean of the *American Veterinary College.*

Dr. E. M. Hunt, Secretary of the *New Jersey State Board of Health.*

Professor Rush S. Huidekoper, Dean of the *Veterinary Department of the University of Pennsylvania.*

William L. Zuill, M. D., D. V. S. of the same institution.

Dr. Salmon, chief of the *Bureau of Animal Industry*, Washington, D. C.

Prof. Chas. B. Michener, of the *American Veterinary College.*

Prof. Smith, Principal of the *Ontario Veterinary College.*

Prof. Law, of *Cornell University.*

Prof. C. P. Lyman, of *Harvard University.*

Prof. D. McEachran, of *Montreal Veterinary College.*

Dr. George Fleming, of London, England.

Prof. James L. Robertson, of the *American Veterinary Colle g* was proposed by Dr. Pierce.

Prof. Baker, Principal of the *Chicago Veterinary College* was proposed by Dr. Voorhees.

Prof. Dr. Leisering, *Geh. Med. R.*, *Germany*, was proposed by Dr. Sattler. All the above gentlemen were unanimously elected to honorary membership.

The objects of the new society, as stated in the certificate of incorporation, are :—"The promotion of fraternal feeling among its members ; the welfare of the veterinary profession in general and of New Jersey in particular ; the advancement of the science and art of veterinary medicine and surgery ; aiming to protect the rights and privilege of practitioners, and to elevate the standard of the profession by scientific intercourse."

President Corlies appointed Dr. Krowl, of Passaic, Dr. DeClyne, of New Durham, Dr. Pocock, of Pennington, Dr. Loblein of New Brunswick, and Dr. Voorhees of Somerville, a committee on By-Laws.

The members decided to hold the next meeting in Newark, either in September or October, when the report of the Committee on By-Laws would be acted upon ; and such other businesss transacted as may come before them at that time. The time of calling the meeting was left in the hands of the President.

WM. HERBERT LOWE, D. V. S.,
*Secretary.*

II. OHIO STATE VETERINARY MEDICAL SOCIETY.—The meeting was called to order by the president, Dr. J. C. Meyer, Jr., of Cincinnati, and Dr. W. A. Labron, of Xenia, occupied the secretary's chair. The roll call revealed the following members present : Drs. J. Yonkerman, and D. P. Yonkerman, Cleveland ; T. Kerr, Urbana ; Walter Shaw, Dayton ; E. R. Barnett, Akron ; C. S. Elliot, Greenville ; F. E. Anderson, Findlay ; J. A. Lee, Lima ; W. H. Gribble, Washington Court House ; W. C. Torrance, Cleveland ; R. G. Holland, Wellington ; Wm. McNaughton, Vienna ; W. C. Daniels, Cardington ; J. D. Fair, Berlin ; N. C. McLain, Jeromeville ; W. C. Fair, Cleveland ; A. H. Logan, Bellefontaine ; J. C. Meyer, Jr., Cincinnati ; J. V. Newton, Toledo ; L. A. Levercool, Norwalk ; A. Tanner, Ashtabula ; R. W. Whitehead, Youngstown ; W. E. Wright, Delaware ; L. D. Blanchard, Canton ; G. W. Butler, Circleville ; T. B. Cotton, Mt. Vernon ; J. Charlesworth, Springfield ; W. F. Derr, Wooster ; F. C. Groff, Massillon ; W. R. Howe, Dayton ; and W. A Labron, Xenia. The president delivered an address during which he spoke of the objects of the association and expressed his pleasure at the large attendance. The committee on veterinary legislation made a report. A bill in the interest of the veterinarians was introduced in the Legislature at the last session, but it was defeated because the lawmakers did not think that there were enough practical veterinarians in the State to do the business. The committee thought it best to petition the Legislature to pass a law preventing anyone from practicing the profession unless he had a diploma. Professor Smith, principal of the Ontario Veterinary college, was present, and made an address. He expressed pleasure in meeting so many of his old friends, not only graduates from his own college, but from others. He was equally well pleased to note the extreme harmony which prevailed among the profession in Ohio. The following new members were then admitted: Drs. W. G. Torrance, Cleveland; R. G. Holland, Wellington; W. H. Gribble, Washington Court House; Wm. McNaughton, Vienna; W. C. Daniels, Cardington; Benjamin C. McLain, Jeromeville; and J. D. Fair, Berlin. At two o'clock in the afternoon an operating session was held at Dr. Fair's veterinary infirmary, and a number of succesful operations were performed. Dr. J. C. Meyer, Jr., performed a delicate operation upon a dog, and Dr. F. E. Anderson treated a case of fistulous withers and removed the tumor. Dr. D. P. Yonkerman removed a melanatic tumor from a dog, and Dr. Butler operated on a ridgling horse. The association convened at 7 o'clock for the purpose of hearing papers, with Dr. Meyer in the chair. The first paper read was by Dr. D. P. Yonkerman. The subject was "Beef Inspection as a Part of Public Hygiene." The paper was exhaustive and treated this important subject in a concise and clear manner.

III. Pennsylvania State Veterinary Medical Association.—The semi-annual meeting was called to order at the Hotel Albemarle, Pittsburg, on September 6th, by the President, Dr. T. B. Rayner.

On the roll call, Drs. T. B. Rayner, Chas. T. Goentner, W. L. Zuill, Jas. B. Rayner, N. Rectenwald, Jos. C. Fly, Geo. B. Rayner, Robt. Gladfelter, Jno. R. Hart, W. Horace Hoskins, answered present. Drs. Myers, Wall and Smith, and Mr. McNeill of Pittsburgh were present as visitors.

After the reading of the minutes of the annual meeting, Dr. Chas. E. Bridge, Phila., Dr. F. A. Wall and Dr. Smith of Pittsburg were proposed for membership.

The Board of Trustees on examination of the credentials of Dr. G. Myers, Allegheny, a graduate of Munich, Bavaria, recommended him favorably and on motion he was elected a member.

Appropriate resolutions relative to the loss sustained by the Association in the deaths of Drs. Michael W. Birch and A. H. Lovette, were reported by the committee and adopted.

The committee on legislation reported the progress of their work and asked for suggestions as to the very best way to completely canvass the State. After many suggestions it was decided to thoroughly district the State and by personal work arouse the people to the value of this bill.

Under the head of new business Dr. Hoskins offered a resolution that a permanent committee on Intelligence and Education be created by the President for the gathering and preserving of all in formation, etc., relative to the progress of the profession in the State.

Dr. Wm. L. Zuill offered a resolution creating a committee on Contagious Diseases or Sanitary Science and Police for the State. This committee to report at each meeting the condition of the live stock of our State.

After a short recess the meeting reconvened, and essays being in order, Dr. W. Horace Hoskins of Philadelphia, read an article on the Differential Diagnosis of Spinal Meningitis, Cerebro Spinal Men ingitis and Azoturea, showing particularly the parts which age, character of animal, mode of onset, history of attack played in distinguishing the diseases. A very interesting and valuable discussion followed, participated in by Drs. Zuill, Jas. B. Rayner, Chas. T. Goentner, Hart and Wall, in which the recent outbreak of cerebro spinal meningitis in New Jersey, came in for much consideration.

After hearing the treasurer's report and the appointing of essayists, the meeting adjourned, to convene in Philadelphia in March, 1888

W. Horace Hoskins,
*Secretary.*

